# MOVE - how to foster European mobility for early career scientists in EV research

**DOI:** 10.20517/evcna.2024.93

**Published:** 2024-12-12

**Authors:** Michael W. Pfaffl

**Affiliations:** Animal Physiology and Immunology, School of Life Sciences, Technical University of Munich, Freising 85354, Germany.

The *EVCNA* Special Issue “EV Insight” summarizes eight contributions that were presented at the first “Mobility for Vesicle Research in Europe” (MOVE) symposium. The event took place at the Auditorium of the Law Faculty of the University of Malaga at the Costa del Sol from October 24th-27th, 2023. It was a great honor for me to serve as the Guest Editor for this Special Issue, together with Dr. Marina Cretich. With profound sadness and sorrow, I have to inform you that Marina passed away unexpectedly this summer, on June 29th, 2024. Unfortunately, she did not live to see the successful completion of this Special Issue “EV Insight”. However, I would like to thank her for her great commitment and scientific expertise in the field of extracellular vesicles (EV) and for putting this Special Issue together. Marina was employed as a senior researcher at the Institute of Chemical Sciences and Technologies of the Italian National Research Council, headed the “Laboratory for the Study of Extracellular Vesicles” since 2020, and was an active member of both the Italian Society of Extracellular Vesicles (EVIta) and the MOVE consortium.

MOVE is an international open consortium of all European national EV societies that got together to promote mobility in EV science and connect research groups across Europe. Their overarching aim is to encourage international communication by organizing European-wide scientific events, hands-on workshops and initializing PhD student travel scholarships between the societies to promote EV research. The MOVE activities are intended to stimulate future collaborations between members in different European laboratories and across different biological, medical, or diagnostic disciplines, taking advantage of geographical proximity and diverse scientific expertise.

The Spanish EV society GEIVEX served as the head organizer of this first MOVE event, supported by the German EV society (GSEV), the British EV society (UKEV) and the EVIta. They spared no effort to organize a fabulous, international symposium with more than 300 international participants, mainly with members from further 16 joining European EV societies, like ASEV, BESEV, BSEVs, CzeSEV, EVIta, FISEV, FSEV, GEIVEX, GSEV, HSEV, NLSEV, NOR-EV, PNEV, PSEV, SrEVs, and UKEV.

For 4 days, the international symposium centered on 13 different sessions on new isolation and MISEV-compliant characterization concepts, clinical or even cosmetic applications of EVs, with a particular focus on their role as intercellular communicators in health and disease, as well as therapeutic targets. Furthermore, novel methods for the identification and development of new EV-related biomarkers at the RNA, DNA, protein, or metabolic level, including holistic and integrative omics technologies, were presented at the symposium. In total, 76 oral presentations and 157 posters were showcased mainly by PhD students or early career EV scientists. An extensive industrial exhibition accompanied the talk and poster sessions, providing the opportunity for participants to have fruitful discussions with industrial partners and find out about new devices and EV-related research products on the market. In the EVCNA Special Issue “EV Insight” at hand, 8 out of 13 submitted manuscripts from participants of the 1st MOVE symposium were published:

(1) The introductory paper is the symposium summary report written by PhD students of my own laboratory (Yu *et al*., 2024)^[1]^. It gives a general overview of all presentations, focusing on the talks with a very short wrap-up of each oral presentation. The meeting review gave special attention to the talks of the five internationally renowned keynote speakers, who contributed overviews about their latest EV projects, scientific findings and how they can be applied in the near future:

“EVs and parasitic diseases: let’s MOVE-on” by Hernando del Portillo;

“Harnessing Nature’s nanoSecrets: Microalgal-derived extracellular vesicles as bio-based nanoparticles for next-level pharmaceutical and cosmetic applications” by Antonella Bongiovanni;

“Convergence of G protein-coupled receptor (GPCR) and extracellular vesicle biology” by Martine Smit;

“Tetraspanins and extracellular vesicles: together and forever” by Fedor Berditchevski;

“Escaping the endosome: overcoming the final barrier for effective drug delivery” by Benjamin Winkeljann.

(2) The second review describes the potential clinical applications of EVs in pancreatic cancer and explores the untapped opportunities from biomarkers to novel therapeutic approaches (Sanchez-Manas *et al*., 2024)^[2]^. The article outlines diverse diagnostic and analytical methodologies used to study and characterize EVs and their role as drivers of tumor development in pancreatic cancer, a highly lethal and metastatic malignancy. Additionally, the review investigates the potential of EVs in liquid biopsies as diagnostic, prognostic, and therapeutic biomarkers for pancreatic cancer. The author concludes that the emerging field of EV research offers great opportunities to improve the diagnosis, prognosis, and therapy of pancreatic cancer. The EV community must continue to unravel the mysteries of EV biology and should strive to develop innovative EV-based technologies to help improve the outcomes for pancreatic cancer patients. Furthermore, it highlights the need to achieve consensus and methodological standards in order to get the maximum benefit out of EVs.

(3) The next contribution to this Special Issue is aimed at the continuous homogenous production of functional and high-quality immortalized mesenchymal stromal cell (MSC)-derived EVs in a hollow fiber bioreactor (Garcia *et al*., 2024)^[3]^. MSC-EVs have been reported to hold great potential as cell-free therapies due to their low immunogenicity and minimal toxicity. In the study, MSC lines from two donors were immortalized (iMSC) and inoculated over 4 weeks into hollow fiber bioreactors and harvested daily. The isolated iMSC-EVs remained viable during the entire culture period, while the MSCs maintained their phenotype during the production phase. The results show that setting up a hollow fiber bioreactor system inoculating immortalized MSC lines facilitates the large-scale, functional, and high-quality production of iMSC-EVs, demonstrating the great potential of this type of iMSC production methodology that could help to standardize EV production with future clinical application.

(4) The following review article focuses on the G protein-coupled receptors (GPCRs) and how these can serve as a gateway to targeting oncogenic EVs (Di Niro *et al*., 2024)^[4]^. Cancer-released EVs show evidence to have a central role as mediators of dysregulated signaling in onco-pathological settings and are seen as a key feature driving cancer progression. GPCRs are the “most druggable” target in almost all areas of medicine, accounting for around 60% of drugs in development and more than a third of all currently approved drugs. In the review, the molecular mechanisms linking GPCRs to EV intercellular communication in cancer settings are described in depth. The authors state that many hurdles still need to be overcome, with a focus on the standardization of large-scale EV production and their incorporation in patient EV treatment regimens or clinical trials. In conclusion, all investigated studies demonstrate that there may be therapeutic benefits in cross-targeting EVs through GPCRs, as they represent nodes at crucial points of the EV life cycle in various oncological settings.

(5) The fifth contribution determines the variability in the characteristics and functionality of platelet lysate-derived extracellular vesicles (pEVs), isolated from different sources of platelet concentrate, through wound healing assays (Amengual-Tugores *et al*., 2024)^[5]^. Isolated pEVs were evaluated by means of protein concentration, Nanoparticle tracking analysis (NTA), transmission electron microscopy (TEM), and flow cytometry using the MACSPlex™ arrays for surface analysis profiling of EV. The functionality of the isolated pEVs was determined in cell culture by metabolic activity and lactate dehydrogenase activity determination and through a wound healing assay. In summary, the outcome showed that there is no need to recruit large numbers of donors, minimizing storage time and regulatory concerns. The use of 5-donor platelet pools for pEV isolation for regenerative applications proves beneficial by maintaining batch-to-batch reliability.

(6) The subsequent review highlights synovial fluid EVs as arthritis biomarkers and an additional value of holistic lipid profiling and integrated omics studies (Varela *et al*., 2024)^[6]^. Lipidomics studies in EV research are currently underrepresented, as well as the potential of EV lipids as biomarkers and their role in enhancing our understanding of EV biogenesis and function. The authors state that lipidomics analysis of EVs has not only proven to be valuable but even necessary for a better understanding of the EV molecular signatures in different pathological contexts. Distinct lipid biomarker signatures can lead to the definition of novel biomarkers for diagnosing and monitoring inflammation, as described in an arthritis study showing the presence of hexosylceramides in EVs during inflammation. Furthermore, the identification of specific combinations of different EV components on various molecular levels (like RNA, DNA, proteins, lipids, or other metabolites) acting together can fuel the definition of composite EV biomarkers. The use of multi-omics integration of these EV datasets can unravel new potentials of composite or integrative biomarker signatures. This may improve the understanding of disease heterogeneity and be applied in the future for diagnosis, disease progression monitoring, and more precise treatment tailoring.

(7) Since the latest coronavirus epidemic, the rise of RNA-based therapies and their application has challenged the limitations of traditional drug treatments. Lipid nanoparticles have emerged as a promising solution for RNA delivery, but endosomal entrapment remains a critical barrier. EVs, on the other hand, overcome endosomal degradation by an endosomal escape mechanism. The presented mini-review describes the endosomal escape mechanisms of EV-based drug carriers over lipid nanoparticle-based drug delivery and explores the lessons that can be applied to lipid nanoparticle design (Hagedorn *et al*., 2024)^[7]^. By understanding the molecular and cellular mechanisms of endosomal escape, we will be able to develop more effective drug delivery vehicles in the future, e.g., based on EVs, enhancing the delivery and efficacy of RNA-based therapies.

(8) Tumor-derived EVs play crucial roles in intercellular communication around the local tumor microenvironment and systemically facilitate tumor progression and metastatic spread. They carry a variety of molecules with bioactive properties, such as nucleic acids, proteins, and metabolites, that trigger different signaling processes in receptor cells and induce, among other downstream effects, metabolic reprogramming. Hence, the final contribution describes the metabolic features of tumor-derived EVs and their analytical challenges during diagnostics, as well as their future opportunities as prognostic tools in cancer management (Espiau-Romera *et al*., 2024)^[8]^. The review presents an in-depth compilation of metabolism-related molecules, e.g., enzymes and metabolites, which are described in cancer-derived EVs and their potential use as cancer biomarkers. The authors further discuss the challenges arising in this rapidly evolving field and conclude that cancer-derived EVs hold great potential, but the field of EV metabolism in cancer is still very young and future joint efforts are needed to bring the metabolomics EV community forward, with a focus on methodology standardization.

In conclusion, this Special Issue “EV Insight” presents eight diverse research topics discussed at the 1st MOVE symposium in Málaga. Overall, the meeting was a highly prosperous event for interactions among the international and young EV scientists from all European EV societies. We eagerly anticipate the following MOVE symposia, which we will organize together. The 2nd MOVE symposium was recently held from October 8th-11th, 2024, at the University of Belgrade, Serbia, hosted by Maja Kosanović from the Department for Immunology and Immunoparasitology. Looking ahead, we are excited for the next MOVE event in 2025 and for the continued publication of Special Issues linked to these meetings.

## DECLARATIONS

### Copyright

© The Author(s) 2024.
